# Expression and distribution of *PPP2R5C *gene in leukemia

**DOI:** 10.1186/1756-8722-4-21

**Published:** 2011-05-06

**Authors:** Haitao Zheng, Yu Chen, Shaohua Chen, Yuzhe Niu, Lijian Yang, Bo Li, Yuhong Lu, Suxia Geng, Xin Du, Yangqiu Li

**Affiliations:** 1 Institute of Hematology, Medical College, Jinan University, Guangzhou, P.R. China; 2 Key Laboratory for Regenerative Medicine of Ministry of Education, Jinan University, Guangzhou, P.R. China; 3 Department of Hematology, Guangdong General Hospital (Guangdong Academy of Medical Sciences), Guangzhou, P.R. China

**Keywords:** *PPP2R5C*, leukemia, gene expression, transcript variant

## Abstract

**Background:**

Recently, we clarified at the molecular level novel chromosomal translocation t(14;14)(q11;q32) in a case of Sézary syndrome, which caused a rearrangement from TRAJ7 to the *PPP2R5C *gene. *PPP2R5C *is one of the regulatory B subunits of protein phosphatase 2A (PP2A). It plays a crucial role in cell proliferation, differentiation, and transformation. To characterize the expression and distribution of five different transcript variants of the *PPP2R5C *gene in leukemia, we analyzed the expression level of *PPP2R5C *in peripheral blood mononuclear cells from 77 patients with *de novo *leukemia, 26 patients with leukemia in complete remission (CR), and 20 healthy individuals by real-time PCR and identified the different variants of *PPP2R5C *by RT-PCR.

**Findings:**

Significantly higher expression of *PPP2R5C *was found in AML, CML, T-ALL, and B-CLL groups in comparison with healthy controls. High expression of *PPP2R5C *was detected in the B-ALL group; however, no significant difference was found compared with the healthy group. The expression level of *PPP2R5C *in the CML-CR group decreased significantly compared with that in the *de novo *CML group and was not significantly different from the level in the healthy group. By using different primer pairs that covered different exons, five transcript variants of *PPP2R5C *could be identified. All variants could be detected in healthy samples as well as in all the leukemia samples, and similar frequencies and distributions of *PPP2R5C *were indicated.

**Conclusions:**

Overexpression of *PPP2R5C *in T-cell malignancy as well as in myeloid leukemia cells might relate to its proliferation and differentiation. Investigation of the effect of target inhibition of this gene might be beneficial to further characterization of molecular mechanisms and targeted therapy in leukemia.

## Background

Molecular genetic aberrations could provide the basis for assays that can predict prognosis of individual patients as well as potential molecular targets for novel therapies [1-3]. The process of malignant transformation in leukemia is complex, and many factors such as abnormal gene expression and mutation, chromosomal aberrations, deregulation of various cellular signaling pathways, and deregulation of epigenetic regulation are involved in the development of leukemia [1]. Therefore, new data regarding molecular genetic aberrations in different types of leukemia are needed for further characterization.

**Figure 1:**
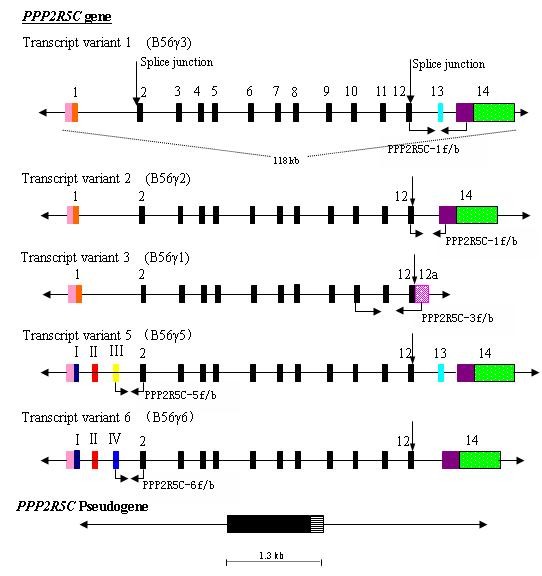
**Genomic organization of *PPP2R5C***. The bars represent the exons, and the lines represent introns. The pink bars are the 5' UTR, the black bars are exons that were identical in all five variants (exons 2-12), the colored bars represent specific exons in different variants, the 3' UTR in different variants is shown with green bars (variants 1, 2, 5 and 6), horizontal-dash-filled pink bars (variant 3), and horizontal-line-filled bars (pseudogene). The coding sequence between exons 2 and 12 and the shown splice junction (downward arrow) were identical across the five splice variants. The location and direction of primers used for amplification of different variants are indicated by arrows [4].

*PPP2R5C *is one of the regulatory B subunits of protein phosphatase 2A (PP2A), which is a major cellular serine/threonine phosphatase that affects the phosphorylation status of many proteins [4]. The *PPP2R5C *gene encodes five differentially spliced variants: B56γ1, B56γ2, B56γ3, B56γ5, and B56γ6 (Figure 1), whereas B56γ4 is identified only in mice. The functional *PPP2R5C *gene locus resides at 14q32.2, whereas a nonfunctional B56γ1 pseudogene *PPP2R5C *is present at 3p21.3 [4,5]. *PPP2R5C *plays a crucial role in cell proliferation, differentiation, and transformation, based on its induction of dephosphorylation of P53 at various residues [6]. It has been reported that the dynamic nuclear distribution of the B56γ3 regulatory subunit controls nuclear PP2A activity and may be responsible for the tumor-suppression function of PP2A [5]. Recently, the alteration of the expression pattern of *PPP2R5C *associated with malignant transformation has been characterized in lung cancer; a *PPP2R5C *mutation, F395C, disrupts B56γ-p53 interaction [7].

Based on our recent finding of a novel chromosomal translocation t(14;14)(q11;q32) that was involved in a gene rearrangement from TRAJ7 to *PPP2R5C *in a patient with Sézary syndrome (data unpublished), in the present study, we further characterized the expression and distribution of five different transcript variants of the *PPP2R5C *gene in leukemia.

## Methods

### Samples

Seventy-seven newly diagnosed and untreated cases of leukemia, including 24 with acute myeloid leukemia (AML); 14 with chronic phase chronic myeloid leukemia (CML); 18 with T-cell acute lymphocytic leukemia/lymphoma (T-ALL); 12 with B-cell ALL (B-ALL); and nine with B-cell chronic lymphocytic leukemia (B-CLL). Twenty-six cases with leukemia in complete remission (CR) (nine with AML-CR, three with B-ALL-CR and 14 with CML-CR) were selected, along with 20 healthy individuals as controls. The samples were collected with informed consent. All procedures were conducted in accordance with the guidelines of the medical ethics committees of the Health Bureau of Guangdong Province, China. Human leukemia cell lines Hut-78, CCRF, Jurkat, Molt-3, Molt-4, K562, NB4, Raji and Daudi were used in the study. The RNA extraction and cDNA synthesis were performed according to the manufacturer's instructions.

### Real-time quantitative RT-PCR (qRT-PCR)

Expression levels of *PPP2R5C *and the reference gene *β2-MG *were determined by SYBR Green I real-time PCR. PCR was performed as our previous description [8]. The 2^(-ΔΔCT) ^method was used to present the data of the genes of interest relative to an internal control gene [8,9]. The sequences of primers used in qRT-PCR were PPP2R5C-for: 5'-GTAATAAAGCGGGCAGCAGG-3' and PPP2R5C-bac: 5'-CAAAGT CAAAGAGGACGCAACA-3' for PPP2R5C gene amplification, β_2_M-for: 5'-CAGCAAGG ACTGGTCTTTCTAT-3' and β_2_M-bac: 5'-GCGGCATCTTCAAACCTC-3' for β_2_M gene amplification.

### Primer design and RT-PCR

To amplify five transcript variants of *PPP2R5C *according the structure of the *PPP2R5C *gene (accession nos. NM_002719.3, NM_178586.2, NM_178587.2, NM_001161725.1 and NM_001161726.1) (Figure 1) [4], we designed 4-pair primers, which covered different exons (Table 1) and confirmed the transcript 3, 5, or 6; however, we were unable to distinguish between transcripts 1 and 5 or transcripts 2 and 6 (Table 2) when positive products were shown in the sample. RT-PCR was performed as in our previous study [10].

**Table 1:** Information on primers used in RT-PCR for *PPP2R5C *segments amplification

primer	sequence	Location	function
PPP2R5C-1f	5'-TGAAAGAACGGGAAGAAGCAT - 3'	1407 bp (12 exon)	Sense primer
PPP2R5C-1b	5'-TGATTGGTATGGCACAGGAAG - 3'	1801 bp (14 exon)	Antisense primer
PPP2R5C-3f	5'-CAGTGACAACGCAGCGAAGAT - 3'	1216 bp (10 exon)	Sense primer
PPP2R5C-3b	5'-ATAAAAACATTCAAGTAACCCTGG-3'	1520 bp (12a exon)	Antisense primer
PPP2R5C-5f	5'-TCCACTTCTTCCTGAGTTGCTG-3'	230 bp (III exon)	Sense primer
PPP2R5C-5b	5'-CTTCTGGGTAAATAGGCTCTGT-3'	472 bp (2 exon)	Antisense primer
PPP2R5C-6f	5'-AGCCTTGTTGCTGTCCCGTCT - 3'	210 bp (IV exon)	Sense primer
PPP2R5C-6b	5'-GTCAAAGAGGACGCAACACTG - 3'	423 bp (2 exon)	Antisense primer

**Table 2:** Amplified PCR products using different primer pairs

Primer pairs	Variant 1 (B56γ3)	Variant 2 (B56γ2)	Variant 3 (B56γ1)	Variant 5 (B56γ5)	Variant 6 (B56γ6)
PPP2R5C1f/PPP2R5C1b	+ 12 + 13 + 14 exons (394 bp)	+ 12 + 14 exons (277 bp)	-	+ 12 + 13 + 14 exons (394 bp)	+ 12 + 14 exons (277 bp)
PPP2R5C3f/PPP2R5C3b	-	-	+ 10 + 11 + 12 + 12a exons (304 bp)	-	-
PPP2R5C5f/PPP2R5C5b	-	-	-	+ III + 2 exons (242 bp)	-
PPP2R5C6f/PPP2R5C6b	-	-	-	-	+ IV + 2 exons (213 bp)

## Results and Discussion

### Expression level of *PPP2R5C *in leukemia

**Figure 2:**
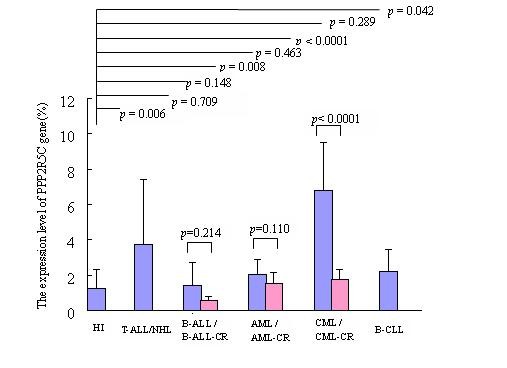
**Expression level of *PPP2R5C *in human leukemic cell line**. A: T-cell lines and healthy CD3^+ ^T cells, B: Myeloid cell lines and B cell lines and PBMCs from healthy individuals.

**Figure 3:**
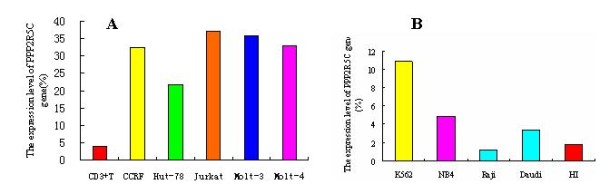
**Expression level of *PPP2R5C *gene in PBMCs from different leukemia patients and healthy individuals (HI)**.

*PPP2R5C *as a potential tumor suppressor plays a crucial role in cell proliferation and differentiation [4]. Based on our recent finding of a novel gene rearrangement from TRAJ7 to *PPP2R5C*, it could be interesting to analyze the expression features of *PPP2R5C *in hematological malignancies. In the present study, we analyzed the expression level of the *PPP2R5C *gene in leukemia samples. In comparison with healthy controls (1.24 ± 1.09), significantly higher expression of *PPP2R5C *was found in the AML (2.06 ± 0.85) (*p *= 0.0076), CML (6.78 ± 2.75) (*p *< 0.0001), T-ALL/NHL (3.73 ± 3.66) (*p *= 0.0062) and B-CLL (2.21 ± 1.22) (*p *= 0.0417) groups (Figure 2). A high tendency toward expression of *PPP2R5C *was detected in the B-ALL group (1.39 ± 1.31); however, the expression was not significantly different from that in the controls (*p *= 0.7089) (Figure 2). The expression level of *PPP2R5C *in the CML-CR group (1.75 ± 0.55) decreased significantly in comparison with the CML group (*p *< 0.0001), but showed no significant difference compared with the healthy group (*p *= 0.2895). Although the expression level of *PPP2R5C *gene decreased in the AML-CR (1.53 ± 0.60) and B-ALL groups (0.54 ± 0.27), there was no significant difference compared with the AML (*p *= 0.1100) and B-ALL groups (*p *= 0.2142) or with healthy controls. Overexpression of *PPP2R5C *was found in T-cell lines like Hut-78, CCRF, Jurkat, Molt-3 and Molt-4, and the expression level was 5-9 times higher than that from healthy CD3^+ ^T cells (Figure 3A). Interesting, the tendency of the expression level of *PPP2R5C *in Raji, Daudi, NB4 and K562 cells was accordant to the results from primary leukemia cells (Figure 3B), which showed higher expression level of *PPP2R5C *in K562 (CML cell line), and lower expression level in B-cell lines (Raji and Daudi).

Mutation, deletion, or abnormal expression of tumor-suppressor genes is frequently identified in leukemia [1,11,12]. To the best of our knowledge, no study has been reported that examined the expression of *PPP2R5C *in leukemia, except for B-CLL. The present results identifying *PPP2R5C *overexpression in most cases of leukemia might have a similar significance in cell transformation. Definitive results for the association of the expression level of *PPP2R5C *with disease progression and outcome could be demonstrated by a comparison of the data for de novo CML and CML in complete remission. In contrast, Fält *et al*. have described that downregulated *PPP2R5C *is related to progressive B-CLL by using the Affymetrix GeneChip technique, and they have concluded that *PPP2R5C *could be a marker of progressive disease in B-CLL. They have compared expression of *PPP2R5C *between stable and progressive B-CLL, but not with a healthy control group [13]. In the present study, we found that expression of *PPP2R5C *was significantly increased in the B-CLL group, similar to AML, CML, and T-ALL; however, we were unable to collect samples from patients in complete remission and from those with progressive disease to compare the expression level and evaluate the significance of *PPP2R5C *detection in B-CLL. Further investigation is needed. Unlike most leukemia cases, the expression level of *PPP2R5C *in the B-ALL group, as well as in B-cell lines did not differ significantly from that in the controls, but it remains unknown whether this was due to the limited number of samples or whether it reflects a true feature of *PPP2R5C *in B-ALL.

### Distribution of *PPP2R5C *transcript variants in healthy individuals and leukemia

**Figure 4:**
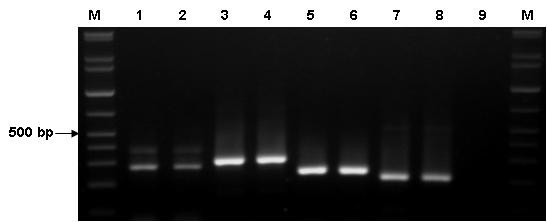
**Results of PCR amplification for *PPP2R5C *gene using different primer pairs**. Lane M: 100-bp DNA ladder; 1 and 2: amplicom using PPP2R5C1f-/PPP2R5C1b primers; small products were 277 bp (12 + 14 exons) and large products were 394 bp (12 + 13 + 14 exons); 3 and 4: amplicom using PPP2R5C3f/PPP2R5C3b primers, the product was 304 bp (10 + 11 + 12 + 12a exons); 5 and 6: amplicom using PPP2R5C5f/PPP2R5C5b primers, the product was 242 bp (III + 2 exons); 7 and 8: ampilcom using PPP2R5C6f/PPP2R5C6b primers, the product was 213 bp (IV + 2 exons); 9: negative control.

It has been reported that five transcript variants of *PPP2R5C *might contribute to the specificity of PP2A [4]. However, little is known about the distribution of different variants in different organs, tissues, and cells, as well as in leukemia cells. Based on the structure of the *PPP2R5C *gene reported from Genbank and previous studies [4], we drew a schematic diagram of the genomic organization of *PPP2R5C *with five transcript variants (Figure 1), designed four primer pairs to amplify different exons, and tried to identify different variants in the same sample. By using PPP2R5C-1f/PPP2R5C-1b, which covered exons 12-14, two expected PCR products were detected. The small one comprised 277 bp containing exon 12 and 14 segments (corresponding to B56γ2 or B56γ6), and the large one comprised 394 bp containing exon 12, 13, and 14 segments (corresponding to B56γ3 or B56γ5). PPP2R5C-3f/PPP2R5C-3b, PPP2R5C-5f/PPP2R5C-5b, and PPP2R5C-6f/PPP2R5C-6b primer pairs covered exons 10 to 12a, exon III to 2, and exon IV to 2, respectively, and the expected PCR products were 304, 242, and 213 bp (corresponding to transcript B56γ1, B56γ5, and B56γ6 respectively) (Table 2 Figure 4). According the structure of transcript variants of *PPP2R5C *gene and the size of the amplicons, we confirmed the B56γ1, B56γ5, and B56γ6 variants. However, using the designed primer pairs, we were unable to distinguish between transcripts 1 and 5 or between transcripts 2 and 6 because PCR products of the same size were amplified by using the PPP2R5C-1f/PPP2R5C-1b, and it could not distinguish these variants using different primer pair combinations, except for whole gene sequencing. B56γ3 (variant 1) and B56γ2 (variant 2) are the frequency variants; therefore, it is thought that they might be expressed when positive PCR products are found. More importantly, using the present methods, we confirmed the expression of B56γ5 and B56γ6, which have been newly identified. Therefore, it could be concluded that all variants can be detected in healthy as well as leukemia samples with a similar frequency and distribution of *PPP2R5C*.

In conclusion, to the best of our knowledge, this is the first description of the expression level of the *PPP2R5C *gene as well as the distribution of *PPP2R5C *transcript variants in PBMCs from different types of leukemia. Overexpression of *PPP2R5C *is a common feature in most types of leukemia; thus, the change in expression pattern might influence the activity of PP2A and could be related to abnormal cell proliferation, differentiation, and transformation. Further research on the downregulation of *PPP2R5C *in leukemia cells is needed to investigate its biological function.

## Competing interests

No potential conflicts of interest and financial disclosure statements except for grants mentioned in the acknowledgements.

## Authors' contributions

YQL contributed to concept development and study design. HTZ performed the real-time PCR, YC performed the RT-PCR, SHC and YZN prepared RNA and cDNA, LJY and BL prepared the PBMCs and collected the clinical data. YHL, SXG and XD were responsible of the patient's treatment and carried out acquisition of clinical data. YQL, HTZ and YC coordinated the study and helped to draft the manuscript. All authors read and approved the final manuscript.
